# A Booklet Self-Help Intervention for People Living with HIV and Depressive Symptoms in Botswana: A Randomized Controlled Trial

**DOI:** 10.1007/s10461-025-04742-7

**Published:** 2025-04-29

**Authors:** Boitumelo Vavani, Nadia Garnefski, Sanne van Luenen, Elise Dusseldorp, Kennedy Amone-P’Olak, Philip Spinhoven, Vivian Kraaij

**Affiliations:** 1 University of Botswana, Gaborone, Botswana; 2 Clinical Psychology, Leiden University, Leiden, The Netherlands; 3 Methodology & Statistics, Leiden University, Leiden, The Netherlands; 4 Faculty of Social Sciences, Department of Psychology, Kyambogo University, Kampala, Uganda

**Keywords:** Depressive symptoms, Self-help, HIV, Randomized clinical trial, Low-and-middle incomecountries

## Abstract

In low- and middle-income countries (LMICs), there is a scarcity of psychological treatment options for people living with HIV (PLWH) with depressive symptoms. Self-help programs for depressive symptoms, in particular, are cost-effective and scalable, and therefore a promising tool in the treatment of depressive symptoms for people in low-resourced countries. This paper presents the results of a study that examined the effectiveness of a guided self-help program in reducing depressive symptoms in PLWH in Botswana. A Randomized Controlled Trial (RCT) was conducted on a sample of PLWH who were screened at HIV treatment centers in Botswana. The RCT had two conditions: an intervention group that received the self-help program with coaching and an attention-only control group. In both groups, a pre-test, post-test, and 3-month follow-up measurement were administered. Patients in the intervention group followed a booklet Cognitive Behavioral Therapy (CBT)-based self-help program. Seventy-two participants were included in the study. The results indicated significantly larger decreases in depressive symptoms in the intervention group than in the attention-only control group, both in the short and longer term, with large effect sizes. In addition, there were significant reductions in anxiety symptoms in the intervention group compared to the control group. The user satisfaction was high. Implementing this low-cost and scalable self-help program in a LMIC such as Botswana is critical in bridging the existing mental health treatment gap. This clinical Trial was registered with the Netherlands Trial registry, number NTR5407on August 23, 2018.

## Introduction

More than three decades since the first case of the Human Immunodeficiency Virus (HIV) was detected in Botswana, the country has one of the highest HIV prevalence rates in the world at 20.8% [1]. Being an upper-middle-income country, many people living with HIV (PLWH) in Botswana have access to free anti-retroviral therapy (ART), which has greatly improved the quality of life and reduced HIV-related mortality [2]. Nevertheless, like many chronic illnesses, HIV progression can be unpredictable, and PLWH may often be required to cope with persistent physical symptoms that may lead to periods of intense emotional distress [3].

Depression is considered one of the most prevalent psychiatric conditions among PLWH [e.g. 4–10]. According to a systematic review and meta-analysis on PLWH living in sub-Saharan Africa (SSA) [5], prevalence rates of depression in PLWH on ART range between 9 and 32% [7, 11]. A more recent systematic review and meta-analysis investigating the burden of depression in outpatient HIV-infected adults in Sub-Saharan Africa, estimated the prevalence of major depression among PLWH at 17% and depressive symptoms at 26% [12]. Based on a few studies conducted in Botswana, the percentages of PLWH who also presented with depressive symptoms are even higher, ranging between 24% and 48% [10, 13, 14].

Depressive symptoms are considered one of the most important and common mental health barriers to medication adherence in PLWH [6, 15, 16]. Additionally, depressive symptoms have been found to further compromise quality of life. For example, many PLWH who also present with depressive symptoms have been found to have poorer health [17], to make less progress with regards to CD4 count, to be at higher risk of committing suicide [18], to generally progress faster to AIDS, and hence show increased rates of mortality [19]. Depressive symptoms have also been shown to be linked to an increase in risk behaviors that may further contribute to the spread of HIV, such as unprotected sexual intercourse, multiple sexual partners, and substance abuse [3]. PLWH are also prone to anxiety disorders. A previous meta-analysis [20] found that 15.5% of HIV patients had anxiety disorders. In Botswana, most research on anxiety amongst PLWH has been conducted among adolescents. Studies show that anxiety is a highly prevalent mental health condition in adolescents and young adults living with HIV in Botswana [21, 22]. Like depression, untreated anxiety is associated with poor medication adherence [23, 24] and has been found to promote risky sexual behaviors, which in turn may lead to increased HIV spread [24]. Given these associations, addressing mental health issues such as depressive symptoms and anxiety among PLWH, in addition to medical care, is critical in HIV prevention and treatment [25–30].

Worldwide, several psychological interventions have been developed to treat depressive symptoms in PLWH and have been found to be effective [e.g. 16, 25–29]. Psychological interventions for depressive symptoms in PLWH were most effective when a CBT approach was used [28]. In addition, research has found larger treatment effects for studies when psychologists were involved as treatment providers [31]. More recently, it was found that psychological treatments could also be effective in treating depressive symptoms in PLWH in Low- and Middle-income Countries (LMICs) [4, 32], including African countries [33–37]. However, it was suggested that interventions should be short and easy to deliver, for example, provided by trained lay counselors [4].

Despite this evidence, there are still many barriers to mental health treatment in Africa for PLWH. These barriers include limited funds, lack of awareness and knowledge of mental disorders, lack of mental health services, a negative attitude towards mental health care [38–41], lack of appropriate screening materials, lack of trained personnel, and lack of psychological treatment programs [13, 14, 41]. In Botswana, for instance, mental health services are delivered though a tiered system with primary care at health posts, secondary care at district hospitals with psychiatric outpatient clinics and tertiary care at the only psychiatric hospital called Sbrana Psychiatric Hospital [42]. When developing psychological programs for PLWH in LMICs, it is important to consider the contextual information of these countries. For example, to overcome personnel barriers, the use of paraprofessionals might be needed in order to reduce the burden on the few psychologists that practice in these countries [4, 8]. To overcome financial and geographical barriers, it has been suggested that a low-cost self-help program would be a promising tool in the treatment of depressive symptoms among PLWH in LMIC [e.g. 16, 43]. Self-help programs are efficient in alleviating symptoms of depression [44] and have the advantage of being cost-effective [45]. Moreover, self-help interventions involve less staff time [46], can be computer-adapted [47, 48], and are suggested to be more suitable in settings short of professional and highly qualified healthcare workers [49].

The present study aimed to fill in the psychological treatment gap for PLWH in SSA, particularly in Botswana, by developing and evaluating a self-help treatment program for PLWH with depressive symptoms, with the potential to reach a high number of people, with low costs and few staff. To do this, an existing program [50] that was empirically based, was selected, as it was shown to be effective, and offered the possibility to adapt it to the needs of PLWH in SSA/Botswana.

This program is called ‘Living positive with HIV’. The program focuses on reducing depressive symptoms among PLWH and is based on principles of CBT. It was developed after a number of empirical studies, first focused on finding the right targets for intervention [51–53] and later on investigating the effectiveness of the program in a Randomized Controlled Trial [16]. There are two versions of the program: a booklet and an online version, with approximately the same content [31, 50]. The program focuses on activation, relaxation, changing maladaptive cognitions, and goal attainment. A randomized controlled trial (RCT) showed that the online program was also effective in reducing depressive symptoms among PLWH in the Netherlands [31].

In the current study, we adapted and evaluated the ‘Living positive with HIV’ program in Botswana. To ensure that the adapted treatment program would address the specific needs of PLWH in Botswana, as an important first step, a pre-study was carried out among 291 PLWH in Botswana [10]. The aims of this pre-study were threefold. First, to confirm that the targets for intervention were the same for PLWH in Botswana as for people in the Netherlands; therefore, it was studied which psychological factors were associated with depressive symptoms. Second, to ask people about their self-reported needs. Third, assess PLWH’s preferences with regard to intervention format and guidance. Regarding the results on the psychological variables, some maladaptive coping strategies were identified, which were positively associated with depressive symptoms, such as rumination, catastrophizing and withdrawal. Also, some more adaptive strategies were identified, which were negatively associated with depressive symptoms, such as positive refocusing and focus on planning [10]. It was concluded that these topics matched the components of the original intervention. Regarding the self-reported needs, the participants expressed a need for help with various mental health problems such as feelings of depression, feelings of anxiety, physical tension, coping with HIV, and finding new goals in life. Regarding participants’ preferences about format and guidance, the findings indicated that a self-help program in booklet format was preferred over an online program and that the participants would like some form of coaching [10].

Based on this information, we tailored the ‘Living positive with HIV’ program to the needs and characteristics of PLWH in Botswana. We adapted a self-help program with coaching in booklet format for PLWH with depressive symptoms in Botswana. Like the original program, the program is based on principles of CBT and includes cognitive-behavioral techniques, stress management, and coping skills. Also, techniques for goal adjustment are included, to help participants to find realistic goals that they want to attain and to develop an action plan to actually attain the goals. The content of the program for Botswana differed from the original program only regarding some of the examples used in the lessons to make them more relevant to the Botswana sample.

In this article, we investigated the effectiveness of this adapted, guided self-help program for PLWH with depressive symptoms in Botswana. An RCT was conducted, in which the intervention group was compared with an attention-only control group which was on a waiting list for the intervention. The effect of the intervention was examined on the short and long term (three months follow-up). Based on previous studies [16, 31], we hypothesized that people who followed the intervention would have a larger reduction in depressive symptoms than the control group. In addition, we investigated the effect of the intervention on anxiety. Also, user satisfaction with the intervention was determined.

## Methods

### Study Design

**Fig. 1:**
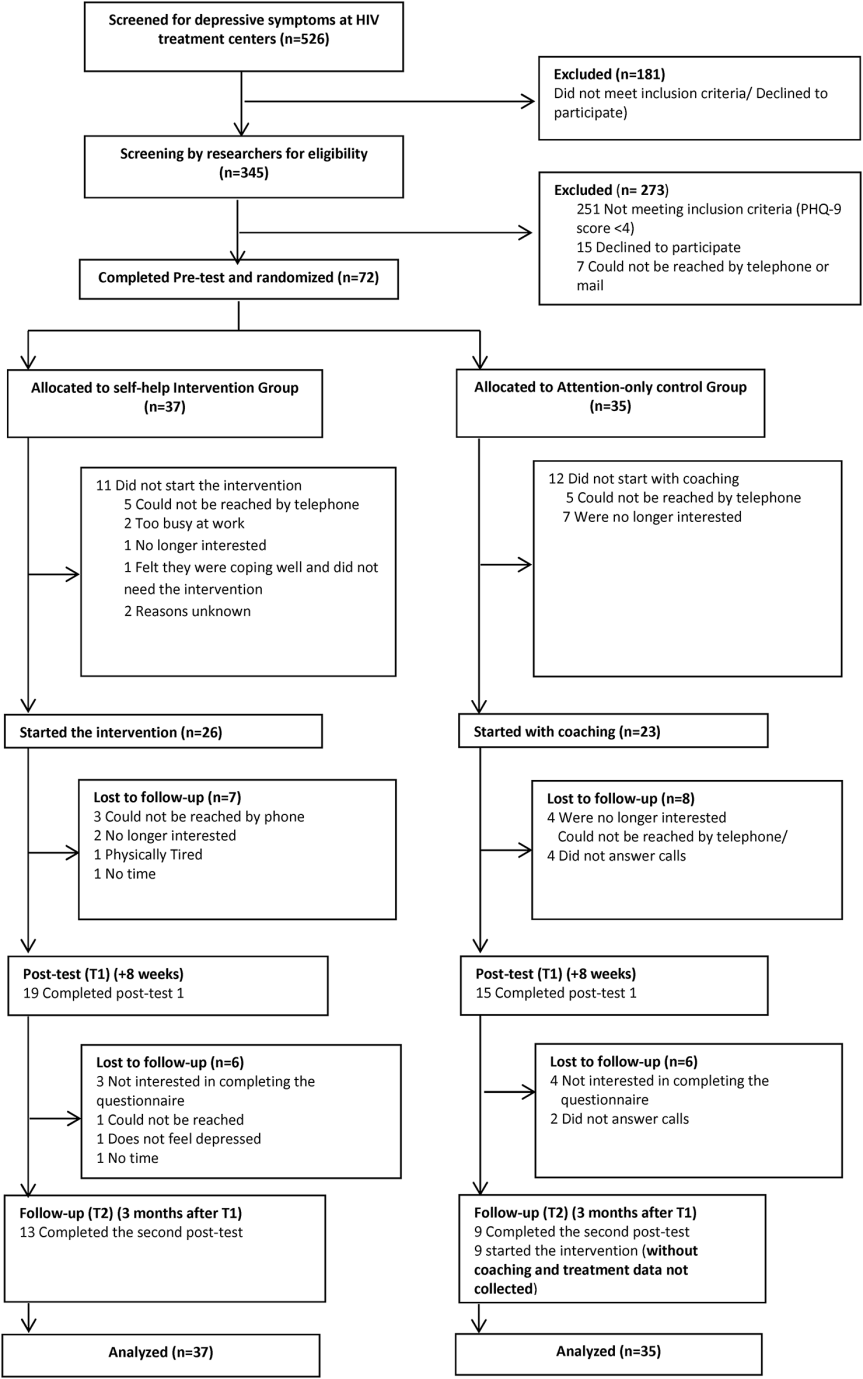
RCT flow chart

An RCT was performed. The trial had two conditions: an intervention group that received the self-help program with coaching and an attention-only control group. In both groups, a pre-test, a post-test and a follow-up measurement were administered. After the waiting period and completion of the follow-up measurement, the attention-only group received the self-help program without coaching. The flow chart of the study is presented in Fig. 1.

### Randomization

Random allocation of participants to the intervention or control group was performed using a stratified random sampling technique. The sample was stratified according to gender and treatment centers. It was important to stratify by gender to ensure approximately equal proportions of males and females in the intervention and control group. Additionally, treatment centers were expected to differ with regard to the number of patients included. Therefore, stratification was meant to ensure that both the intervention and control groups in large and small treatment centers had approximately the same number of patients in both conditions.

Random number tables were used to allocate participants to either group, and these were computer generated and produced by an independent researcher. Randomization was done in permuted blocks of six. The independent researcher entered the numbers into an Excel file which concealed the randomization until after the participants had been part of the trial. The randomization was set-up such that the words (intervention and control) on the Excel file were made white, hiding the condition to be allocated from the main researcher, to minimize bias. During the allocation - after the pre-test- the words were made visible by the main researcher. The coaches and participants were informed of the treatment allocation after the pre-test.

### Participants

The inclusion criteria for participation in the study were (i) being HIV positive and receiving medical treatment in an HIV treatment center, (ii) presenting with mild to moderate depressive symptoms (that is, a score higher than 4 and lower than 20 on the PHQ-9; see measurements), (iii) being aged 18 years and older, (iv) having sufficient knowledge of the Setswana or the English language, and being available for the next eight weeks to work on the intervention. Exclusion criteria were: (i) severe cognitive impairments (clinical judgment by medical staff and researchers), (ii) being in the first six months post HIV-diagnosis, and (iii) suicidality (as determined by a score higher than 1 on the suicide item of the PHQ-9). Those who scored within the severe depressive symptoms range (≥ 20) or showed suicide ideation as measured by the PHQ-9, were referred for face-to-face psychological or psychiatric treatment or to a medical doctor at the treatment center.

### Procedure

Six HIV treatment centers, spread across the country, had agreed to help with the recruitment, by screening and selecting participants. In the current study, data presented was collected from one treatment center (Princess Marina Hospital) due to challenges in screening and referral that were encountered in other study sites. Despite this, the Princess Marina Hospital is the largest hospital in the country [54] and a referral hospital serving individuals from across the country.

We started by providing information about the study, screening guidelines, and screening forms to the treatment centers. We also advertised the study at all participating HIV treatment centers. A poster with information about the study was placed at all study sites. Additionally, the researchers were given an opportunity to introduce the study to potential participants during morning prayers at the treatment centers.

Initial screening of patients who consented was conducted by either nursing consultants or the researchers when the patients arrived for their regular visits to the HIV treatment center. The Patient Health Questionnaire-2 (PHQ-2) [55] was used for this. Patients had to score at least 1 to be eligible for further screening by the researchers. These patients were provided with written information about the study and permission was requested to provide the researchers with the patient’s contact information (telephone number or email). Only patients who volunteered and consented to participate were assessed for eligibility. Subsequently, a second and more elaborative screening took place by the researchers by telephone. In this more elaborate screening, the Patient Health Questionnaire-9 (see measurements) was administered. Patients eligible for participation were invited to participate and were asked to give consent by text message to the researchers. Subsequently, a coach was allocated to each eligible participant to provide support and motivation for the duration of the program. Subsequently, participants completed the pre-test (T0) and were randomly assigned to the intervention or control (attention only) group.

The standard of care for all participants is such that the participants consult with a general practitioner once every three months to six months depending on the length of time since HIV diagnosis and on the response to medication. PLWH also visit the pharmacy for a refill of their HIV medication, either, monthly, quarterly or every six months, also depending on their response to treatment. Those who default on treatment or have a poor response are followed up and sometimes referred for counseling provided by HIV lay counselors.

### Measurement Moments

Recruitment was conducted between January 2019 and March 2020 and participants were enrolled in batches. The pre-test (T0), post-test (T1) and post-test 2 (T2) were administered by the researchers by a standardized telephone interview. Questions were read out aloud exactly as they appeared on the standardized questionnaire. The answer categories for each section were also read out aloud and the participants were asked to write them down for easy reference. Researchers could repeat questions at the request of the participant.

Participants completed the post-test (T1) 8 weeks after the start of the intervention and subsequently post-test 2 (T2) 3 months later. After the second post-test, participants in the control group received the intervention.

### Booklet Self-Help Intervention

The booklet self-help intervention employs Cognitive Behavioral Therapy (CBT) and stress management techniques and is based on self-regulation and stress-coping theories. Based on the CBT model, the focus of the intervention is on changing maladaptive thoughts, behaviors and adapting life goals. The content of the self-help program contains the following main components: activation, relaxation, changing maladaptive cognitions, and the attainment of new personal goals. This content was covered over six lessons that were to be completed in 6 to 8 weeks. The content included a combination of psychoeducation (e.g. a lesson on the vicious circle of negative thoughts, emotions, behaviors, and physical reactions), assignments (e.g. creating a plan for the continuation of the relaxation exercises), and some activities (e.g. finding and changing a negative thought). Participants were expected to work on the program 1–2 h every week. Participants were asked during the weekly calls about the last lesson that had been completed, whether they read the text and explanation in the lessons, and how often they performed the exercises since the last time they completed a questionnaire. These questions were asked to gauge if participants were able to adhere to the intervention timelines and requirements.

The first lesson was an introduction to the program and focused on activation. Participants were asked and encouraged to think of a small concrete new activity to perform. Throughout the program, participants were encouraged to continue thinking about small, concrete new activities to perform. Lesson 2 was on physical relaxation. Participants were taught to do some relaxation exercises that they continued with for the rest of the program. Participants could choose between a body check exercise that was provided in the booklet or follow a YouTube link for a voice recording by either a male or female voice. The third lesson focused on identifying and challenging negative thinking patterns and replacing them with more helpful thoughts. In lesson 4, participants learned strategies to stop unpleasant thoughts by applying a counter-conditioning technique. In lesson 5, participants were helped to find new, meaningful, concrete, and time-bound life goals. They were guided to work on attaining their goals. The last lesson focused on getting self-confidence to achieve their goals. Participants learned to challenge negative thoughts that prevented them from pursuing their goals. An overview of the key lessons from the program was given at the end. The booklet was available in English and Setswana.

### Coaching

All coaches were trained in coaching and motivational interviewing techniques. The study required close monitoring of depressive symptoms; therefore, we decided on having coaches that had obtained at least a Bachelor’s degree in Psychology, had completed a clinical course during their education program, and had been taught communication skills, interview techniques, and treatment strategies such that they could better coach and monitor participants and advise those who needed attention or referral. Additionally, we expected that coaches with a degree in psychology are trained in communications skills and would therefore be able to use MI communication skills. The researchers and a licensed psychologist supervised the coaches through weekly meetings in the first month of the study and biweekly meetings thereafter.

At the beginning of the program (for the intervention group) or waiting period (for the control group), the coaches telephoned the participants.

In the intervention group, the coaches introduced and explained technical aspects of the program and stimulated the participants’ motivation by motivational interviewing (MI) and general conversational techniques. MI is an evidence-based approach to stimulate behavior change [56, 57]. A weekly telephone call was scheduled for approximately 15 min. During the telephone call, the coaches enquired about the progress of the participant, problems encountered and offered support and motivation and encouraged the participants to continue working on the program. No formal psychotherapy was provided to the participants by the coaches. The coaches kept detailed notes on every interaction with participants. A spreadsheet was used to record every step taken by coaches, decisions made and content of the conversations with the participants.

The maximum coaching period was six weeks, at which point the participant was expected to have completed the program. If the participant had not completed the program after six weeks, they were allowed to carry-on with the program, however, the coaching stopped.

In the control group, all participants also had a coach but only minimal support was provided for a maximum of six weeks. The weekly telephone calls between coaches and participants in the control condition were scheduled for approximately five minutes. During the phone call, the coaches used motivational interviewing (MI) and general conversational techniques to prevent participants’ dropout.

### Ethical Precautions

Coaches monitored the depressive symptoms of all the participants on a weekly basis by asking how the participants were doing. A participant could be referred to a medical doctor, their general practitioner, or the HIV treatment center in case they were showing severe depressive symptoms (PHQ-9 > 20) or suicidal ideation. None of the participants needed a referral. Additionally, adverse events were monitored through weekly calls. Participants were also encouraged to report any adverse events immediately and relatedness to the intervention. Psychological distress was assessed through participant interviews at pre-specified intervals and monitored by the study team.

### Measurements

The primary outcome was depressive symptoms, and the secondary outcome was anxiety symptoms. The questionnaires (available in both English and Setswana) also included several other secondary outcomes, which are outside the scope of this article [see 58]. In addition, questions regarding satisfaction with the intervention were included. More specifically, the following measures were included in the present study.

#### Demographic Variables and Other Background Information

Background information was assessed at pre-test (T0). First, demographic variables and personal information were assessed (e.g. gender, age, level of education, etc.). In addition, data with regard to past episodes of depressive and anxiety symptoms, any psychological/psychiatric treatment received for the symptoms and physical health was gathered. Information regarding the HIV infection was also collected, such as date of HIV diagnosis, CD4 cell count, viral load, medication use, adherence to medication, and how satisfied they were with their HIV treatment.

#### Patient Health Questionnaire-9 (PHQ-9)

The PHQ-9 was used to measure the severity of participants’ depressive symptoms at screening, pre-test, post-test 1, and post-test 2 [59]. The questionnaire has nine items measured on a 4-point scale ranging from 0 (not at all) to 3 (nearly every day). The total score is obtained by adding the scores of the nine items and may range from 0 to 27, with the following cut-offs: score of ≤ 4 indicating minimal or no depressive symptoms, 5–9 mild depressive symptoms, 10–14 moderate depressive symptoms, 15–19 moderately severe depressive symptoms and 20–27 severe depressive symptoms. The scale has been widely used for measuring depressive symptoms and has good psychometric properties [59]. In addition, the PHQ-9 is often used with PLWH and has been found to be highly reliable [60]. In Botswana, previous studies support the validity of the PHQ-9 [58, 61] and in phone interview format [62]. In the present study, an alpha reliability of 0.87 was found.

#### For Initial Screening Only: Patient Health Questionnaire-2 (PHQ-2)

The PHQ-2 [59] was used for the initial screening of depressed mood in potential participants at the HIV treatment centers (see procedure). The scale is made up of the first two items of the PHQ-9, measured on a 4-point scale ranging from 0 (not at all) to 3 (nearly every day). A total score is obtained by adding the scores of the two items and may range from 0 to 6, with higher scores indicating more depressive symptoms. A score of 1 or higher is considered indicative of potential depressive symptoms. The scale has shown good psychometric properties [60].

#### Generalized Anxiety Disorder-7 (GAD-7)

The GAD-7 [61] was used to measure participants’ symptoms of anxiety at pre-test (T0), post-test (T1) and post-test 2 (T2). The scale has 7-items that use a four-point scale ranging from 0 (not at all) to 3 (nearly every day). The total score is obtained by adding the scores of the 7 items and may range from 0 to 21. Higher scores indicate more symptoms of anxiety. The following cut-offs are used: score of ≤ 4 indicating minimal or no anxiety symptoms, 5–9 mild, 10–14 moderate, and 15 and higher severe anxiety symptoms. The scale has been shown to have adequate psychometric properties [61]. A previous study [58] has reported good psychometric properties for the GAD-7 in a sample of PLH in Botswana. In the present study, an alpha reliability of 0.90 was found.

#### User Satisfaction

The following questions were asked at post-test (T1) to determine participants’ satisfaction with the self-help program and their coach: (1) If you would evaluate the self-help program with a grade, what number, mark or grade would you give the program? on a scale between 0 (very bad program) and 10 (very good program); (2) Would you recommend other persons to follow this program (answer categories: certainly not, maybe, certainly)?; (3) If you would evaluate your coach with a grade, what number, mark or grade would you give your coach on a scale between 0 (very bad support from the coach) to 10 (very good support from the coach)? The first two questions were only answered by participants from the intervention group. Both groups answered the question about the coach.

### Statistical Analysis

Power analysis according to the methods of Brysbaert [62] and Xi, Pennell, Andridge, and Paskett [63] resulted in a target sample size of *N* = 70 (*N* = 35 per group) based on the following assumptions: a medium to large effect size (Cohen’s *d* = 0.60), a reliable outcome measure (Cronbach’s α > 0.80), a pre-post correlation of 0.50, a minimum power of 0.80, and a two-sided significance level α of 0.05.

Data was analyzed with SPSS software (version 29). We carried out Intention to Treat Analysis (all randomized participants were analyzed) [64]. We used a two-tailed alpha of 0.05 for significance testing. Data analysis was based on pre-test, post-test 1 and post-test 2 scores. Screening scores were not included in the data-analysis.

To investigate baseline differences between conditions at the pre-test, we used chi-square tests, t-tests and ANOVA’s.

To investigate differences between conditions (referred to as Group, coded with 1 = intervention and 0 = control condition) with regard to changes in symptoms of Depression (PHQ-9), and Anxiety (GAD-7), Longitudinal Multilevel Regression Analyzes (LMRA) were applied. Time was treated as a categorical variable in the analysis. The pre-test was used as reference variable (coded with 0). Dummy variables were created for post-test 1 and post-test 2 (both coded with 1), representing short-term and long-term change, respectively (referred to as Short-term and Long-term). As histograms indicated that the distributions of the two dependent variables were all positively skewed, violating the assumptions of LMRA, log transformations were applied for these analyzes. Fixed effects in the analysis were Short-term and Long-term and the interaction of Short-term x Group and Long-term x Group. Significant interaction effects imply that the conditions differ in their mean change in symptoms at short-term and long-term. The variable Group was excluded as simple effect in the analysis, implying that the pre-test means for both conditions are constrained to be equal in the analysis. Due to the RCT design, this is a plausible constraint and recommended by Cohen [65]. A random intercept model was used. The Variance Components covariance structure was selected, and maximum likelihood estimation was used to estimate the model coefficients.

Furthermore, Cohen’s *d* was used to compute the effect size. It was obtained by calculating the difference in mean predicted values (from the LMRA model) between intervention and control condition and dividing this value by the pooled standard deviation. Cohen’s *d* of 0.20 was considered a small effect, 0.50 a medium effect, and 0.80 and higher a large effect [66].

To assess whether pre-post differences in PHQ-9 and GAD-7 scores reflected ‘true’ clinical significance, the Jacobson and Truax (JT) approach was applied [67, 68], which included three steps. In step 1, it was determined whether the individual change scores changed from a ‘clinical’ to a ‘normal’ status after intervention. For both the PHQ-9 and GAD-7 the cut-off point of 5 was used (see measurements). A change was considered a clinical improvement if the individual’s score – from pre-test to post-test – improved from a score higher than (or equal to) the cut-off to a score below the cut-off point. Clinical deteriorations were determined likewise. In step 2, it was determined whether the individual change scores could not be attributed to measurement errors. For this purpose, the Reliable Change Index (RCI) was calculated. The formula for the RCI is: [post – pre-test score] / se_dif (standard error of difference). [*Se_dif was obtained from the formula*: $\:SD\sqrt{2}\sqrt{1-{r}_{xx}}$* with SD referring to the Standard Deviation and r*_*xx*_*referring to the Cronbach’s alpha at pre-test*]. Individual RCI values lower than − 1.96 were assumed to reflect a significant improvement (p < .05), RCI values higher than + 1.96 were assumed to reflect a significant deterioration. In step 3, the information of the previous two steps was combined. A change score could only be called a ‘true’ clinical and significant recovery when both criteria were met: The score had both changed from a clinical’ to a ‘normal’ status after the intervention or waiting period, and the RCI was lower than − 1.96. These steps were taken for the individual PHQ-9 and GAD-7 change scores at post-test 1 and post-test 2. Recovery could only be tested for participants that scored above the cut-off at pre-test.

Numbers needed to treat were calculated using the percentage of participants who met the criteria for ‘true’ clinical and significant recovery. The per-protocol analysis sample was used to calculate clinically significant changes and numbers needed to treat.

The user satisfaction data were analyzed using descriptive statistics. An independent *t*-test was used to test differences between the intervention and control groups regarding the grade given to the coach.

## Results

Figure 1 presents the study flow chart. A total of 526 PLWH were screened for depressive symptoms, of whom 345 were screened for eligibility by the researchers. Seventy-two participants completed the pre-test (T0) and were randomly assigned to the intervention group (*n* = 37) or the control group (*n* = 35). Of the 37 participants assigned to the intervention group, 26 (70%) started the intervention, 19 (51%) completed the first post-test (T1) and 13 (35%) completed the second post-test (T2). Twenty-three (66%) of those assigned to the control group started with the coaching, 15 (43%) completed the first post-test (T1), and 9 (26%) completed the second post-test (T2). Participants dropped out of the trial for various reasons as indicated in the study flow chart (see Fig. 1). Nine participants from the control group started with the intervention after the second post-test (T2).

### Background Characteristics

Table 1 gives the figures for the background characteristics of participants in the intervention and control group at baseline. Females made up 67.0% of the sample. The mean age of the respondents was 48.1 years (SD = 9.6). The majority (95.8%) of the respondents were taking antiretroviral therapy. The average length of time since the HIV diagnosis was 14.4 years (SD = 5.44). A history of depression and anxiety was reported by 34.7% and 16.7% of the participants, respectively. In terms of educational background, 41.7% of the participants had no/primary education, and the rest (58.3%) had managed secondary education and further. About half (51.4%) of the participants were unemployed. To investigate differences between conditions, we used chi-square tests, t-tests and ANOVA’s. The only significant difference at baseline was found for employment status; in the intervention group, significantly more people were employed than in the control group.

**Table 1:** Demographic and other background characteristics of participants in the intervention and control group at pre-test

Characteristic	Total sample (*n* = 72)	Intervention group (*n* = 37)	Control group (*n* = 35)
Age in years, mean(SD)	48.1(9.63)	46.7 (10.51)	49.6(8.47)
Gender, n (%)			
Male	24(33%)	13(35%)	11(31%)
Female	48(67%)	24(65%)	24(69%)
Nationality, n (%)			
Motswana	71(99%)	36(100%)	33(97%)
Zimbabwean	1(1%)	0	1(3%)
Education, n (%)			
No/Primary education	30(41.7%)	15(40.5%)	15(42.9%)
Secondary education and further	42(58.3%)	22(59.5%)	20(57.1%)
Employment, n (%)			
Yes	35(48.6%)*	23(62.2%)	12(34.3%)
No	37(51.4%)	14(37.8%)	23(65.7%)
Marital status, n (%)			
Married/cohabiting	37(52.1%)	21(58.3%)	16(45.7%)
Not married	34(47.9%)	15(41.7%)	19(54.3%)
History of depression, n (%)			
Yes	25(34.7%)	12(32.4%)	13(37.1%)
No	47(65.3%)	25(67.6%)	22(62.9%)
History of anxiety, n (%)			
Yes	12(16.7%)	4(10.8%)	8(22.9%)
No	60(83.3%)	33(89.2%)	27(77.1%)
Time since HIV diagnosis (in years), mean(SD)	14.42 (5.44)	14.95 (4.72)	13.85 (6.16)
AIDS Diagnosis, n (%)			
Yes	2 (2.9%)	1 (2.7%)	1 (3%)
No	68(97.1%)	36(97.3%)	32(97%)
CD4 count, mean(SD)	626.8 (274.6)	693.2 (286.9)	548.2 (241.2)
Antiretroviral therapy, n (%)			
Yes	69(95.8%)	37(100%)	32(91%)
No	3(4.2%)	0	3(9%)

Values are the mean (SD), or *n* (%). Some percentages are rounded off, therefore, totals may not equal 100

*Significant difference between intervention and control groups

### Differences Between Intervention and Control Group in Depression and Anxiety Scores Over time

Table 2 presents the observed baseline and post-test mean scores for the PHQ-9 and GAD-7. No baseline differences were found between conditions, as was tested by *t*-tests.

**Table 2:** Observed pre-test and post-test mean scores for PHQ-9 and GAD-7

Primary/secondary outcome measures	Total (M (SD))	Intervention group (M (SD))	Attention-only control group (M (SD))
PHQ-9			
Pre-test	7.71 (6.51) [*n* = 71]	7.84 (6.45) [*n* = 37]	7.58 (6.68) [*n* = 34]
Post-test 1	3.00 (3.43) [*n* = 34]	1.63 (1.86) [*n* = 19]	4.73 (4.20) [*n* = 15]
Post-test 2	2.41 (3.13) [*n* = 22]	0.85 (0.99) [*n* = 13]	4.67 (3.81) [*n* = 9]
GAD-7			
Pre-test	7.63 (5.81) [*n* = 72]	7.41 (15.90) [*n* = 37]	7.86 (5.78) [*n* = 35]
Post-test 1	3.26 (3.17) [*n* = 34]	2.21 (2.07) [*n* = 19]	4.60 (3.85) [*n* = 15]
Post-test 2	3.68 (4.43) [*n* = 22]	1.92 (1.98) [*n* = 13]	6.22 (5.78) [*n* = 9]

These are untransformed mean scores. In the LMR analyses we used the log-transformed scores

Table 3 presents the results of the LMRA analyzes (after log transformation of the dependent variables) on the differences between the intervention and control group in changes of depression (PHQ-9) and anxiety symptoms (GAD-7) over time. Pre-post effects were calculated for (short-term) post-test 1 and (longer-term) post-test 2.

**Table 3:** LMR analyses: differences between intervention and control group in changes of depression and anxiety symptoms over time

Measure and time point	General time effect				Time by group effect			
	b	SE	t	*p* value	b	SE	t	*p* value
PHQ-9 (log-transformed)								
Intercept (pre-test mean)	7.71							
From pre-test to post-test 1	− 0.21	0.09	– 2.35	0.021*	− 0.26	0.11	– 2.25	0.027*
From pre-test to post-test 2	− 0.19	0.11	– 1.68	0.097	− 0.43	0.14	– 3.04	0.003**
GAD-7 (log-transformed)								
Intercept (pre-test mean)	7.63							
From pre-test to post-test 1	− 0.19	0.08	– 2.30	0.024*	− 0.21	0.10	– 2.02	0.046*
From pre-test to post-test 2	− 0.11	0.10	– 1.08	0.284	− 0.34	0.13	– 2.63	0.010**

*: *p* ≤ .05; **: *p* ≤ .01; ***: *p* ≤ .001

With regard to the differences between conditions in mean change in symptoms at the short-term (Table 3; time by group), significant differences were found for both measures; decreases (improvements) in depression and anxiety scores at the short-term were significantly larger in the intervention group than in the control group.

Regarding the changes in symptoms at the long-term, once more, significant differences were found between conditions for both measures. Also on the longer term, the intervention group experienced significantly larger decreases in depression and anxiety scores than in the control group.

### Effect Sizes for the (LMRA) Differences Over Time Between Intervention and Control Group

Both outcomes had large effect sizes for the differences between the intervention and control groups, both in the short and long term. Regarding post-test 1, the following effect sizes were found (Cohen’s *d*): *d* = 0.76 for the PHQ-9 and *d* = 0.81 for the GAD-7. Regarding post-test 2, the effect sizes were: *d* = 1.38 for the PHQ-9 and *d* = 1.04 for the GAD-7.

### The Clinical Significance of Individual Changes

Regarding the Jacobson & Truax criteria for ‘true’ clinical significance of the individual change scores, the following results were found.

With regard to the PHQ-9 change scores from pre-test to post-test 1: Of the 19 participants in the intervention group that completed the first post-test, 11 participants had an initial pre-test score in the ‘clinical’ range. For 10 of these 11 participants the individual score from pre-test to post-test improved to the ‘normal’ range (step 1). For 63.6% (7/11) this reflected ‘true’ significant improvement with an RCI < -1.96 (step 2). This equals an improvement of more than 6.51 points, reflecting ‘true’ clinical change rather than measurement error (step 3). In the control condition, 9 of 14 participants that completed the first post-test had a pre-test score in the ‘clinical’ range. Two of them (22.9%) improved to a score in the ‘normal’ range at post-test with an RCI < -1.96.

With regard to the PHQ-9 from pre-test to post-test 2: 13 participants in the intervention group completed the second post-test; 9 of them had an initial pre-test score in the ‘clinical’ range; All 9 scored in the ‘normal’ range at the second post-test, of whom 77.7% (7/9) had an RCI < -1.96, reflecting ‘true’ clinical improvement. In the control group, of the participants that completed the second post-test (*n* = 9) and also had a pre-test score in the ‘clinical’ range (*N* = 6), 33.3% (2/6) improved to the ‘normal’ range with an RCI <-1.96.

With regard to the GAD-7 from pre-test to post-test 1: 19 participants in the intervention group completed the first post-test; 11 of them had an initial pre-test score in the ‘clinical’ range; eight of the 11 (72.7%) scored in the ‘normal’ range at post-test and had an RCI < -1.96, reflecting ‘true’ clinical improvement. In the control group, of the participants that completed the first post-test (*N* = 15) and also had a pre-test score in the ‘dysfunctional’ range (*N* = 10), 30.0% (3/10) improved to the ‘normal’ range with an RCI <-1.96.

With regard to the GAD-7 from pre-test to post-test 2: 13 participants in the intervention group completed the second post-test; 8 of them had a pre-test score in the ‘clinical’ range; Seven of the 8 scored in the ‘normal’ range at the second post-test and also had an RCI < -1.96 (87.5%), reflecting ‘true’ clinical improvement. In the control group, of the participants that completed the second post-test (*N* = 9) and also had a pre-test score in the ‘clinical’ range (*N* = 6), 33.3% (2/6) improved to the ‘normal’ range with an RCI <-1.96. There were no significant deteriorations observed in any of the groups.

Regarding the numbers needed to treat for post-test 1: these were 4 and 6 for the PHQ-9 and GAD-7 respectively. For post-test 2, numbers needed to treat were: 3 (PHQ-9) and 4 (GAD-7) (no table).

### User Satisfaction

Most participants were satisfied with the intervention. On a scale of 1–10, their mean grade for the self-help program was 8.26 [SD = 1.15; *n* = 19]. Sixteen participants (84.2%) would definitely recommend the intervention to others, 2 (10.5%) would maybe recommend it, and 1 (5.2%) would not recommend the intervention. The mean grade they gave for their coach (scale 1–10) was 9.05 [SD = 0.19; *n* = 19] in the intervention group against 7.47 (1.36; *n* = 15) in the control group (*t* (32) = 4.07; *p* <.001). No adverse events were reported.

## Discussion

The aim of this study was to investigate whether working on a guided self-help program could effectively reduce depressive symptoms in PLWH in Botswana. The results confirmed that significantly larger decreases in depression symptoms were found in the intervention condition than in the attention-only control condition, both in the short and longer term, with large effect sizes. Also, anxiety symptoms significantly decreased in the intervention group. For a considerable part of the sample, a ‘true clinical recovery’ was realized, according to the JT approach. The user satisfaction was high.

This is the first RCT demonstrating that it is possible to achieve a significant reduction in depressive symptoms through a self-help program in booklet format for PLWH in SSA/Botswana. That is an important finding, given the urgency to treat depressive symptoms in PLWH [10, 13, 14] in combination with the lack of mental health services in Botswana and the sub-Saharan region [14, 38–41]. The urgency lies in the knowledge that depressive symptoms have not only been shown to be highly associated with poorer physical health and quality of life [17], but also with increased sexual risk behaviors [3], poorer HIV medication adherence [15], and higher AIDS and mortality rates [18, 19].

Several strengths, limitations and challenges should be noted. One of the strengths was the strong study design of an RCT comparing an intervention group with an attention-only control group. In addition, well-validated questionnaires were used as outcome measures. The results regarding the PHQ-9 and GAD-7 all confirmed that the program was able to reduce symptomatology in the short and longer term, providing confidence in the results. Another strength was that the self-help program in booklet format was based on an existing, evidence-based self-help program with proven effectiveness, which had been adapted to the specific needs of PLWH in Botswana, on the basis of a pre-study/needs assessment carried out among the target group [10]. The high user satisfaction probably reflected the efforts to make the program suitable for this specific group. Another strong point is that the intervention was in booklet format, so that it could reach many people at the same time with minimal stigma. In the current study the self-help program was available in English and Setswana, but it could also easily be translated into other languages for use in other countries. In the study, we added coaching to the program, which was highly appreciated by the participants, both in the intervention and in the control group. By offering some (minimal) coaching in the control condition, we had an active control condition. Because pure waiting-list control conditions might inflate the effects of interventions, we tried to reduce that by using a control condition that was more active than a pure waiting-list control condition. In addition, using an active control group was an effort to have lower drop-out in the control condition and to meet the ethical standards of care.

The first limitation of the study was the high drop-out at post-test 1 and 2 which reduced the power for the analyzes. However, this had been captured by using Longitudinal Multilevel Regression Analyzes, which had the big advantage that all available participants with baseline data could be included in the analyzes for the estimation of model parameters. A second limitation was that the inclusion of participants was based on screening scores, whereas data-analysis was based on pre-test scores. These scores could differ due to the time in between the measurements. Future studies should keep the time between screening and start of the study as short as possible. Another limitation was that only self-report was used to assess depressive symptoms. This is somewhat overcome by the fact that the self-report measures were all well-validated and widely used with evidence-based cut-off scores. Although a diagnosis of depression was not an inclusion criterion in the current study and interviews would have been too time-consuming for the present study, obtaining the clinical diagnoses would undoubtedly have been a relevant addition to our study. Because of the small sample size, our findings might not be generalizable to all PLWH in Botswana or other SSA countries. Another limitation of our study was the short follow-up period which could have prevented an accurate assessment of the long-term effects of the intervention, potentially underestimating both benefits and harms, as significant outcomes might only become apparent after a longer duration of time following treatment. Additionally, the use of coaches with a bachelor’s degree could have limited the study’s external validity. This could lead to some treatment sites not adopting the intervention in case of limited individuals with a bachelor’s degree to provide coaching. Furthermore, another study limitation related to the difficulty in reaching some participants by phone such that they could not start the intervention for this reason. Therefore, potentially this led to exclusion or reduced participation. Another limitation of the study related to differences in employment status between conditions, which could be a potential confounding factor, given its association with depressive symptoms. Additionally, we did not include adherence to the intervention in our data analysis since we did not include a measure for it. Future studies should use a measure to include this information.

The good news is that we have an intervention with proven effectiveness in reducing depression and anxiety symptoms. Moreover, it is a booklet that can be made easily available to anyone who needs it (printed or as pdf, online), regardless of time, place and presence of care providers. The challenge, however, is to get it to the target group in question and to motivate people to start and complete the intervention. Probably it is most feasible to link implementation of the intervention to the hospitals, the doctors and nurses on site, as almost all PLWH regularly visit the hospital to collect their medication. While our coaches had a degree in psychology, it might be useful to consider training the psychiatric nursing staff and lay-counselors to provide the coaching and conduct motivational interviewing, given the small number of individuals with a degree in psychology in Botswana. For future research, we recommend a longer follow-up period which would ensure accurate assessment of the long-term effects of the intervention. We recommend only having this longer follow-up for the intervention group, due to ethical reasons [69]. Additionally, we recommend that future studies explore the use of other professionals or lay conselors as coaches to address the issue of external validity. We also recommend that other methods of contacting participants be explored, such as email, or posts to ensure that patients are reached when needed.

In conclusion, the self-help program ‘Living positive with HIV’ was found to be effective in reducing symptoms of depression and anxiety in PLWH in Botswana. The self-help program and the coaching were positively evaluated. The results suggest that many people of the target group could be helped by carefully implementing the program, not only in Botswana, but also in the surrounding countries and probably also in other LMIC countries.
